# Sow Estrus Detection Based on the Fusion of Vulvar Visual Features

**DOI:** 10.3390/ani15182709

**Published:** 2025-09-16

**Authors:** Jianyu Fang, Lu Yang, Xiangfang Tang, Shuqing Han, Guodong Cheng, Yali Wang, Liwen Chen, Baokai Zhao, Jianzhai Wu

**Affiliations:** 1 Agricultural Information Institute, Chinese Academy of Agricultural Sciences, Beijing 100081, China; 821012410534@caas.cn (J.F.);; 2 Key Laboratory of Agricultural Blockchain Application, Ministry of Agriculture and Rural Affairs, Beijing 100081, China; 3 State Key Laboratory of Animal Nutrition and Feeding, Institute of Animal Science, Chinese Academy of Agricultural Sciences, Beijing 100193, China; 4 Beijing Jiahua Breeding Pig Co., Ltd., Beijing 101299, China

**Keywords:** machine learning, intelligent farming, sow estrus detection, YOLOv11

## Abstract

Under large-scale farming conditions, automated sow estrus detection is crucial for improving reproductive efficiency, optimizing breeding management, and reducing labor costs. Conventional estrus detection relies heavily on human expertise, a practice that introduces subjective variability and consequently diminishes both accuracy and efficiency. Failure to identify estrus promptly and pair animals effectively lowers breeding success rates and drives up overall husbandry costs. In response to the need for the automated detection of sows’ estrus states in large-scale pig farms, this study proposes a method for detecting sows’ vulvar status and estrus based on multi-dimensional feature crossing. The method adopts a dual optimization strategy: First, the Bi-directional Feature Pyramid Network—Selective Decoding Integration (BiFPN-SDI) module performs the bidirectional, weighted fusion of the backbone’s low-level texture and high-level semantic, retaining the multi-dimensional cues most relevant to vulvar morphology and producing a scale-aligned, minimally redundant feature map. Second, by embedding a Spatially Enhanced Attention Module head (SEAM-Head) channel attention mechanism into the detection head, the model further amplifies key hyperemia-related signals, while suppressing background noise, thereby enabling cooperative and more precise bounding box localization. To adapt the model for edge computing environments, Masked Generative Distillation (MGD) knowledge distillation is introduced to compress the model while maintaining the detection speed and accuracy. Based on the bounding box of the vulvar region, the aspect ratio of the target area and the red saturation features derived from a dual-threshold method in the HSV color space are used to construct a lightweight Multilayer Perceptron (MLP) classification model for estrus state determination. The network was trained on 1400 annotated samples, which were divided into training, testing, and validation sets in an 8:1:1 ratio. On-farm evaluations in commercial pig facilities show that the proposed system attains an 85% estrus detection success rate. Following lightweight optimization, inference latency fell from 24.29 ms to 18.87 ms, and the model footprint was compressed from 32.38 MB to 3.96 MB in the same machine, while maintaining a mean Average Precision (mAP) of 0.941; the accuracy penalty from model compression was kept below 1%. Moreover, the model demonstrates robust performance under complex lighting and occlusion conditions, enabling real-time processing from vulvar localization to estrus detection, and providing an efficient and reliable technical solution for automated estrus monitoring in large-scale pig farms.

## Simple Summary

Timely and accurate estrus detection in sows is critical for improving reproductive efficiency and reducing labor costs in modern pig farming. However, traditional detection methods rely on human observation, which is labor-intensive and prone to error. In this study, we developed a lightweight, non-contact detection system that automatically identifies sow estrus status based on the image features of the vulvar area. The proposed model combines multi-scale feature fusion and channel attention mechanisms to improve detection accuracy and robustness under real-world farm conditions. The system is designed for edge deployment and achieves real-time performance on embedded devices. Field tests demonstrate that it provides reliable estrus monitoring, offering a practical solution for intelligent breeding management.

## 1. Introduction

With the intensification of the livestock industry, improving sows’ reproductive efficiency has become a key factor in ensuring a stable meat supply [1,2]. Although synchronization techniques are widely used in large-scale pig farms for production management, they can induce group stress reactions that may lead to instances of non-pregnancy and adversely affect animal welfare [3,4]. Traditional estrus detection primarily relies on human observation of sows’ standing reflexes, vulvar swelling, and mucosal coloration; however, this method has inherent limitations [5,6]: inspection is labor- and time-intensive, making round-the-clock monitoring challenging [7]. Additionally, the results are highly subjective due to differences in individual expertise [8]. Furthermore, existing automated devices often rely on contact sensors, which suffer from complex installation and high maintenance costs [9], thereby restricting the widespread adoption of the technology.

Accurate detection of the sow estrus cycle directly impacts reproductive efficiency. Studies indicate that the sow estrus cycle averages 21 days, with the effective estrus period lasting only 40–60 h [10], and ovulation typically occurring during the final two-thirds of the estrus period [11]. To achieve optimal conception rates, artificial insemination must be precisely timed within 0–24 h before ovulation [9], placing stringent requirements on the timeliness of detection. Failure to promptly recognize the estrus state may result in the sow’s annual piglet production (PSY) falling below the economic threshold of 20–30 piglets [12]. Under such conditions, the risk of culling sows due to substandard reproductive performance increases significantly, accompanied by reduced farrowing rates and prolonged non-productive days (NPDs), which greatly elevate the operational costs of pig farms [13]. Therefore, there is an urgent need to develop an objective, real-time, and non-contact estrus detection device to support modern sow estrus monitoring.

The advancement of computer vision technology has provided new approaches for estrus detection. Previous studies have attempted to analyze behavioral features using deep learning—such as detecting erect ears during estrus [14] or changes in activity levels [15,16]—but these features are often affected by individual variations, making them unstable across different breeds and growth stages. In contrast, changes in the vulva, as a direct physiological indicator of estrus, have stronger biological foundations and greater potential for quantitative analysis [17]. Existing vision-based detection methods have primarily focused on texture, size changes, or temperature distribution. For example, Seo et al. used vulvar texture analysis to determine estrus state [18]; however, due to complex environments and contamination in practical pig farms, the detection accuracy was only about 70%. Zhang et al. employed YOLOv4 to measure vulvar dimensions and convert them into millimeter-level measurements, achieving an Accuracy of 97% for size detection [19]. Nevertheless, due to the difficulty of obtaining reliable vulvar measurements, this metric is hard to promote as a stable indicator for estrus detection. Xu et al. combined LiDAR-acquired 3D information with behavioral data to achieve a detection accuracy of 95.2 ± 4.8% [20], but the high hardware costs and complex maintenance limited the method’s practical application. Similarly, Xue et al. used infrared thermography to monitor temperature changes [21], but contamination on the sow’s body interfered with the image quality and detection stability, and the high cost was prohibitive for many farms. Although computer vision technology offers new ideas for sow estrus detection, existing methods still face several challenges in practical applications [22]. Moreover, current mainstream end-to-end deep learning methods, although capable of directly outputting estrus judgments, suffer from a “black box” nature that makes their decision processes difficult to interpret; differences among pig groups, environments, and lighting conditions can easily lead to a reduced generalization ability and compromised detection stability. These challenges stem primarily from the lack of explicit modeling of key morphological cues and poor robustness in region localization. Most existing methods rely solely on RGB-based appearance features without incorporating multi-scale spatial semantics or attention-based refinement, leading to the inaccurate detection of the vulvar area under complex lighting and occlusion conditions.

This study focuses on the efficient detection of sows’ vulvar features and proposes a lightweight estrus detection method based on an improved YOLOv11 model. Unlike approaches that directly output estrus results, our method incorporates a feature selection mechanism within the detection framework: YOLOv11 is first used to accurately locate the vulvar region, and then biological features are integrated for classification. To enhance both the detection accuracy and robustness, key features such as the aspect ratio and saturation are employed. The aspect ratio, as a normalized morphological parameter, quantifies changes in vulvar shape while mitigating the effects of individual differences and variations in camera distance; saturation, on the other hand, enhances the model’s sensitivity to blood flow changes during estrus and reduces the influence of varying lighting conditions on color features. The two independent models are optimized separately and then combined with machine learning and physiological insights, resulting in detection outputs that are more interpretable and better suited to complex farming environments.

**Figure 1:**
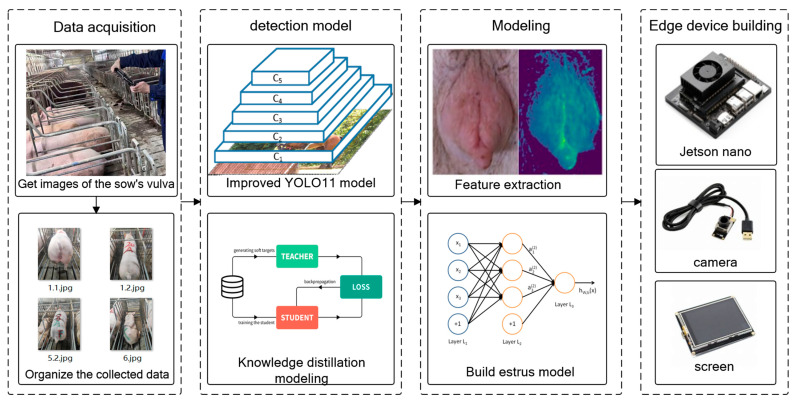
Experimental procedure.

In terms of model optimization, this study introduces a BiFPN-SDI feature fusion module to improve multi-scale feature extraction and employs a SEAM-Head channel attention mechanism to enhance the expression of key features. To reduce computational costs, MGD knowledge distillation is used to compress the model, reducing its size by 91% to 3.96 MB while optimizing the detection efficiency with a per-frame detection time of 18.87 ms, enabling real-time operation on low-cost edge devices such as the Jetson Nano. Experimental results indicate that even under complex conditions, the proposed method maintains high detection accuracy. The device assists caretakers in quickly identifying suspected estrus animals during routine inspections, effectively reducing missed mating opportunities and improving breeding efficiency. It is especially suitable for farms with limited manpower. Compared with existing YOLOv4-based detection methods, our approach not only improves adaptability, but also reduces the computational burden, making the estrus detection system more viable for practical deployment in large-scale pig farms. Figure 1 shows the technical process of this research.

## 2. Experimental Data and Methods

### 2.1. Experimental Site and Dataset Construction

#### 2.1.1. Experimental Area

**Figure 2:**
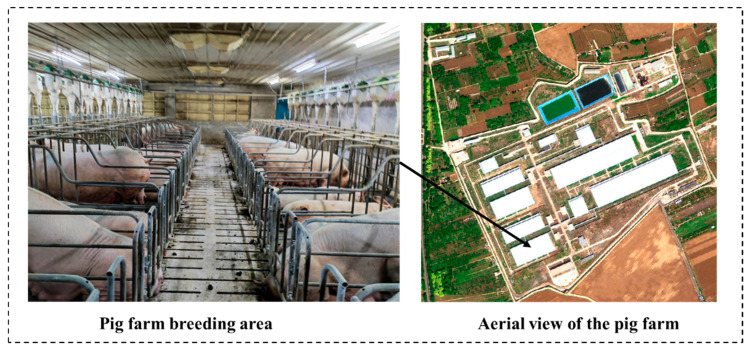
Experimental site.

All the experimental data for this study were collected in December 2024 and April 2025 at Jia Hua Breeding Pig Company in Pinggu District, Beijing, China. The experimental site is located at approximately 117°12′42.89″ E and 40°09′24.40″ N, in a region characterized by a temperate monsoon climate, which is well-suited for sow breeding and raising. The pig farm raises 70% Landrace pigs, 20% Large White pigs, and 10% Duroc pigs. The single pig house covers an area of 400 square meters, with a total of 300 multiparous sows, which are raised in separate pens. Since the pig farm is managed in a standardized manner, its lighting conditions and environmental factors are constant and there is no off-site interference, making it very suitable for estrus-related experiments. This study collected estrus characteristic data from approximately 300 sows across four different time periods. Figure 2 shows the research environment.

#### 2.1.2. Data Collection and Dataset Construction

This study collected estrus characteristic data from approximately 300 sows across four different time periods. The images for this study were acquired using a 13-megapixel ultra-wide-angle camera on a mobile phone (resolution: 3072 × 4096). Data collection took place from 8 December to 24 December and from 8 April to 28 April, covering the estrus status of sows in both winter and summer. As the farm implements synchronized estrus management, a total of four batches of estrus data were collected, each representing a complete estrus cycle. To align with the practical requirements for estrus checking in production, images of the sow’s vulva were captured twice daily—once from 10:00 to 11:30 AM and again from 3:00 to 4:30 PM. The images were taken from a distance of 30–60 cm from the sow’s vulva, at eye level, to ensure clear image quality. All images were captured under the farm’s standard LED lighting environment (18 h light/6 h dim), resulting in largely uniform illumination; therefore, light condition stratification was not necessary. A total of 2000 samples were collected, with each sow being imaged 6–8 times per session to guarantee the accuracy and consistency of the vulvar features.

**Figure 3:**
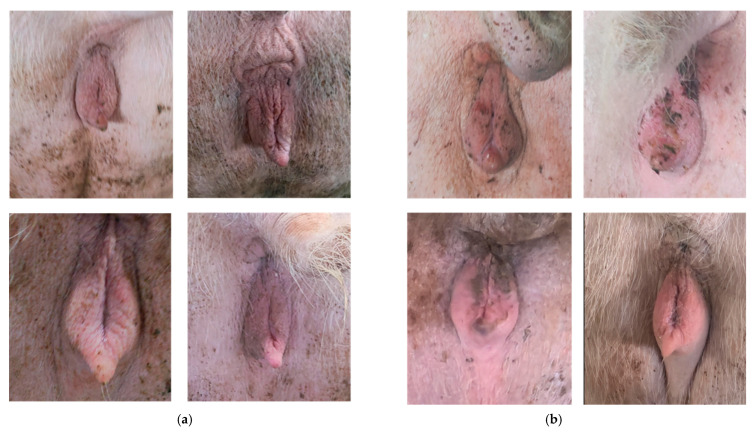
Data collection and classification. (**a**) Sow vulva in non-estrus state. (**b**) Sow vulva in estrus state.

Due to issues with shooting angles, some images exhibited poor quality. To ensure data quality, the original images were filtered in this study. Using Python, images were sorted based on lighting intensity and sharpness, and those with lighting conditions significantly different from the actual scene or with low clarity were removed. Additionally, images with targets that were too small or captured from improper angles were manually excluded. In the end, 1400 images were retained. Next, the vulvar regions were annotated, with the annotation strictly limited to the lateral and upper edges of the labia as well as the area below the labia and above the clitoral region, to support subsequent aspect ratio calculations. Finally, the dataset was divided into training, validation, and testing sets in an 8:1:1 ratio. Figure 3 shows the collected images of the sow’s vulva in different states. This study used a stratified random split at the individual sow level, so that images of the same sow never appeared in more than one subset. Finally, some of the excluded images were retained for future external evaluation.

All bounding boxes and estrus labels were annotated by one person according to the above standards, and to ensure the consistency of the labels, another researcher re-checked 140 randomly selected images. No major differences were found.

### 2.2. Vulva Region Detection Model Based on Improved YOLO

**Figure 4:**
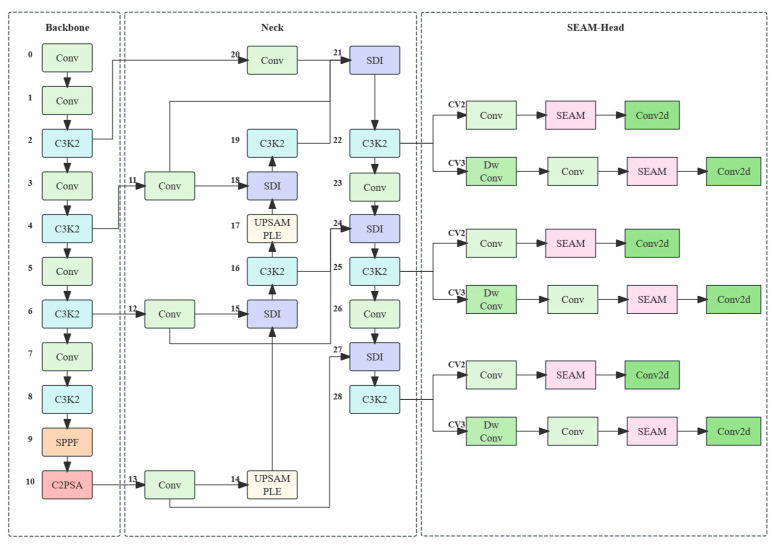
Schematic diagram of the improved YOLOv11 algorithm.

In order to achieve a faster and more accurate segmentation of the sow’s vulvar region, this study improves the YOLOv11 network architecture (see Figure 4). Specifically, a multi-level feature fusion module (SDI) is integrated with the classic weighted bidirectional feature pyramid network (BiFPN) to form a novel Neck structure. In addition, a SEAM module is incorporated into the detection head to enhance the network’s attention capability. The final model, named YOLOv11-BiFPN-SDI-SEAMHead, achieves improved detection accuracy and adaptability.

#### 2.2.1. Experimental Platform

The model is built on the PyTorch 1.6 deep learning framework. The experimental platform is equipped with an Intel Core™ i7-11700k processor (Intel Corporation, Santa Clara, CA, USA) and an NVIDIA GTX 3090 (NVIDIA Corporation, Santa Clara, CA, USA) graphics card with 24 GB of memory, running on Ubuntu 18.04 with Python 3.8, CUDA 11.4, OpenCV 4.5.1, and other necessary deep learning environments.

#### 2.2.2. BiFPN-SDI Improved Feature Pyramid

The sow’s vulvar region is relatively small and exhibits complex edge features, which can result in the extraction of irrelevant features during model training, ultimately affecting the model’s performance [23]. This is particularly problematic when the vulva is swollen or exhibits lesions. To address this issue, the BiFPN-SDI module is integrated into the network to improve the localization and segmentation accuracy of the vulvar region.

**Figure 5:**
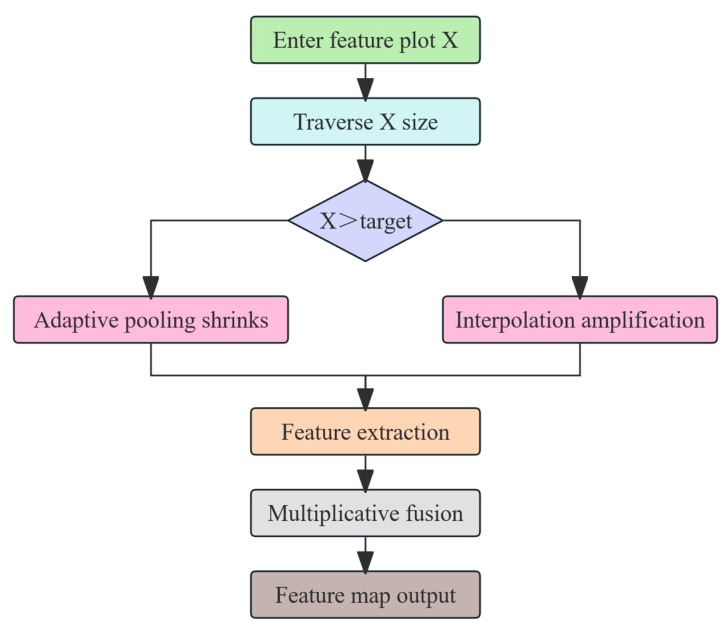
Schematic diagram of the SDI algorithm.

The BiFPN (Bidirectional Feature Pyramid Network) is an efficient multi-scale feature fusion network that incorporates a unique weighted fusion mechanism [24]. This mechanism optimizes the feature fusion process by assigning weights to each input feature, allowing the network to emphasize features that carry more informative content [25]. In this study, SDI (Selective Decoding Integration) is employed to enhance the feature fusion mechanism based on the BiFPN by introducing a selective decoding strategy that makes better use of multi-scale features [26]. Unlike the original weighted fusion in the BiFPN, SDI incorporates an attention mechanism, making the fusion process more flexible and precise [27] (see Figure 5). The improved BiFPN-SDI module, with its dynamic feature selection capability provided by the attention mechanism, highlights critical features for tasks such as sow vulva detection while reducing background interference.

(1) $$F_{output}=\sum_{i}\frac{\alpha_{i}}{\sum_{j}\alpha_{j}}\times Att\left(F_{i}\right)$$

$F_{i}$ represents the feature maps at different scales; $Att\left(F_{i}\right)$ denotes the feature selection weights generated by the attention module, which further enhance the effective features; $\alpha_{i}$ is a learnable global weight used to dynamically adjust the importance of each feature layer; and $\frac{\alpha_{i}}{\sum_{j}\alpha_{j}}$ is the normalized weight (i.e., *α_i_α_i_* divided by the sum overall *α_j_α_j_*).

#### 2.2.3. SEAM-Head Attention Mechanism Optimization

Due to issues such as fecal contamination and occlusion via confinement bars during detection, the accuracy of identifying the vulvar region and detecting estrus can be adversely affected [28]. To address these challenges, this study introduces the SEAM-Head to optimize the Detect component of the neural network. The SEAM (Spatially Enhanced Attention Module) enhances the classification accuracy and improves the extraction of features for small target detection. Moreover, the optimization of the Detect component focuses on the direct output stage, directly improving the precision of the bounding box boundaries, the ability to handle occlusions, and the accuracy of state recognition, thus simplifying the detection process [29].

**Figure 6:**
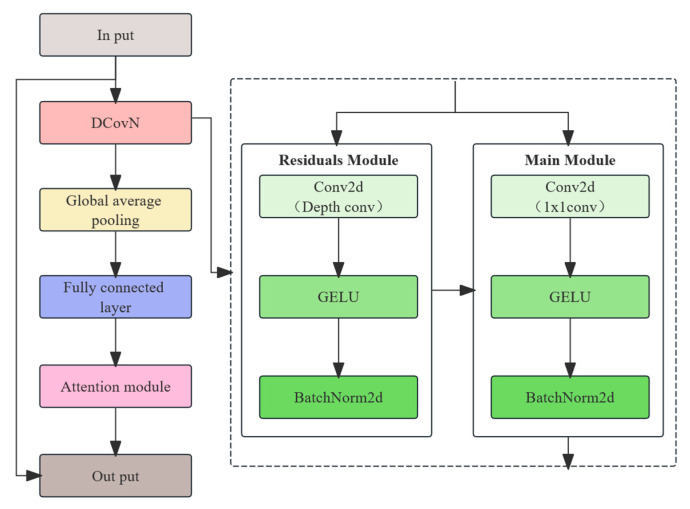
Schematic diagram of the SEAM-Head algorithm.

The SEAM-Head is an attention mechanism module originally designed for semantic segmentation, typically used to improve feature representation in segmentation models. In the detection branch, after the convolution layers, the core objective of the SEAM module is to enhance the model’s focus on important features by employing a channel attention mechanism combined with multi-scale feature fusion, thereby improving the overall accuracy (see Figure 6).

SEAM uses the GELU activation function to enhance the non-linear expressive capabilities. The mathematical formula for GELU is as follows:

(2) $$GELU\left(x\right)=0.5x\left\{1+\tan h\left[\sqrt{\frac{2}{\pi }}\left(x+0.044715x^{3}\right)\right]\right\}$$

Additionally, BatchNorm2d (batch normalization) is applied to normalize the input, thereby enhancing the model’s generalization ability. The core concept of BatchNorm2d is to normalize the input data of each layer so that it has zero mean and unit variance [30]. This normalization helps to prevent gradient vanishing or exploding and improves the training speed and stability, thereby ensuring Accuracy and Precision. Here, $x_{i}$ represents the i input sample; $\mu_{C}$ is the mean of channel C; $\sigma_{C}^{2}$ is the variance of channel C; ϵ is a small number used to avoid division by zero; and γ and β are the scaling and shifting factors, respectively.

(3) $$y_{i}=\gamma \left(\frac{x_{i}-\mu_{C}}{\sqrt{\sigma_{C}^{2}+\epsilon }}\right)+\beta$$

#### 2.2.4. Model Complexity Calculation

The BiFPN-SDI neck processes four backbone feature maps through five fusion stages, comprising three 2 × 2 down samplings and two nearest-neighbor up-samplings; each stage is gated by an SDI module with four channel attention heads, adding 24.6 k parameters per gate. The full sequence contributes 0.12 M parameters and 3.1 M FLOPs, with a latency estimate of 0.40 ms. The Detect_SEAM head up-samples P5 by ×2, aligns its channels with P4, and concatenates it with P3–P4–P5 before passing through two 1 × 1 convolutions. No additional channel or spatial attention is applied. In a 128-channel, single-class setup, this adds 0.066 M parameters and 1.4 M FLOPs, with an estimated latency of 0.28 ms (see Table 1).

All latency and memory statistics were measured on the deployment target, an NVIDIA Jetson Nano (quad-core Cortex-A57 CPU @ 1.43 GHz, 128-core Maxwell GPU, 4 GB LPDDR4, and JetPack 5.1.1). The batch size was fixed to 1 to reflect real-time streaming. Timing was averaged over 500 consecutive frames (640 × 640) with Python time wrappers and CUDA events; memory was recorded with Tegra stats.

Together, the two modules introduce ≈ 0.19 M parameters and ≈4.5 M FLOPs—only 3.7% of the student model’s capacity—while keeping the overhead low enough for real-time deployment in pig house conditions.

#### 2.2.5. Model Knowledge Distillation

Although the optimized model achieves high accuracy, its complexity and large training weights increase the computational load and inference time, which is not conducive to deployment and practical application. Knowledge distillation can compress the model by transferring the information from a complex, large model to a smaller one [31]. During training, the small model is able to learn the weight information and target details of the large model [32]. Knowledge distillation is often used to accelerate inference or reduce storage requirements while retaining the performance of the larger model as much as possible. In order to deploy the model on edge devices later, this study applies knowledge distillation to the optimized model.

**Figure 7:**
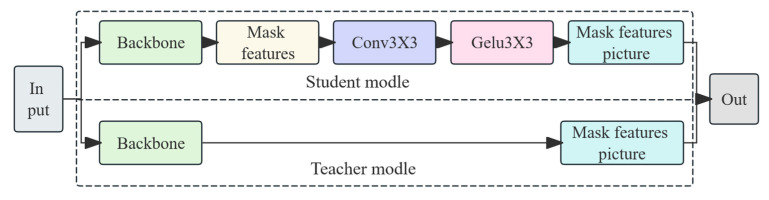
Schematic diagram of MGD knowledge distillation.

Several knowledge distillation approaches have been widely adopted in lightweight model transfer, such as logit-based distillation (SoftTarget) and intermediate feature-based methods (FitNet). These methods weaken the feature extraction and representation ability of shallower detection networks [33], which poses additional challenges. In this study, we employ MGD (Mask Generative Distillation) to transform the imitation task into a generation task—enabling the student model, with its relatively weaker features, to generate features that resemble the stronger features of the teacher model [34]. Compared to simpler alternatives, MGD introduces stronger spatial supervision, better aligns with the region-focused nature of our detection task, and enhances background suppression. Although it imposes a slightly higher computational overhead during training, it leads to improved transfer efficacy for downstream deployment in edge environments. This choice is further motivated by the need for robust small-object detection in occluded and low-contrast conditions, where MGD helps to maintain localization precision and detection reliability. Specifically, the student features are randomly masked; the mask is multiplied with the adjusted feature map to obtain the masked feature, which is then processed through two successive 3 × 3 convolution layers and an activation module to generate a new feature map. This new feature map is then used for knowledge distillation against the teacher network’s feature map (see Figure 7). The formula for the random masking is as follows:

(4) Mi,jl=0, if Ri,jl<λ1, Otherwise

$R_{i,j}^{l}$ is a 0–1 random number; i,j represent the horizontal and vertical coordinates of the feature map, respectively; and λ denotes the hyperparameter for the masking rate. The corresponding mask is applied to the student network’s feature map, and the masked student network then attempts to generate the teacher’s feature map for the unmasked pixels, as shown in the following formula:

(5) $$G\left[f_{align}\left(S^{l}\right)\times M^{l}\right]\to T^{l}$$

Here, $f_{align}$ denotes adaptive adjustment. The goal of MGD is to make the features of the student network approach those of the teacher network as closely as possible, and the distillation formula is designed as follows:

(6) $$L_{dis}\left(S,T\right)=\sum_{l=1}^{L}\sum_{k=1}^{C}\sum_{i=1}^{H}\sum_{j=1}^{W}\left\{T_{\left(k,i,j\right)}^{l}-G\left[f_{align}\left(S_{\left(k,i,j\right)}^{l}\right)\times M_{i,j}^{l}\right]\right\}$$

Here, *L* is the total number of distillation layers; *C*, *H*, and *W* represent the number of channels, the height, and the width of the feature map, respectively; and *S* and *T* denote the features of the student and teacher networks, respectively.

YOLOv11n, compared with YOLOv11m, has fewer channels, which reduces the model size and computational load. Therefore, this study employs YOLOv11n as the student model for knowledge distillation. The best.pt file obtained from the YOLOv11-BiFPN-SDI-SEAMHead model is used for the teacher model weights, while YOLOv11n.pt is used for the student model weights for distillation.

### 2.3. Sow Estrus Detection Based on Vulvar Features

#### 2.3.1. Estrus Vulvar Features and Feature Data Extraction

The changes in the sow’s vulvar features during estrus are primarily induced by elevated estrogen levels. Physiological studies have shown that during estrus, vasodilation of the vulvar mucosa leads to congestion and a significant increase in the epidermal hemoglobin concentration, a phenomenon that visually manifests as an enhanced red saturation in the vulvar region [35]. At the same time, the congestion-induced tissue edema causes the vulva to swell, shifting its morphology from elongated toward a rounder shape. Based on this biological mechanism, this study establishes a comprehensive multi-feature extraction method.

The bounding box obtained from target detection is normalized in terms of length, width, and center coordinates. This step effectively eliminates interference from hair, feces, and other background elements, ensuring that subsequent estrus detection focuses solely on the target region. For color feature extraction, the detected region is converted from the BGR to HSV color space. Compared to the RGB space, the HSV space’s color separation property allows its S channel (Saturation) to directly represent color purity and to be robust against variations in illumination intensity [36]. The red saturation is calculated using a dual-threshold masking method. Based on the distribution of the visible spectrum, two red-sensitive intervals in the HSV space are defined: a low-red interval $\left(H\in \left[0,10\right],S\geq 50,V\geq 50\right)$ that covers deep red tones, and a high-red interval $\left(H\in \left[160,180\right],S\geq 50,V\geq 50\right)$ that captures magenta hues. After merging the masks, the mean pixel value of the S channel is extracted as the saturation indicator.

(7) $$RedSaturation=\frac{1}{N}\sum_{i=1}^{N}S_{i}$$

**Figure 8:**
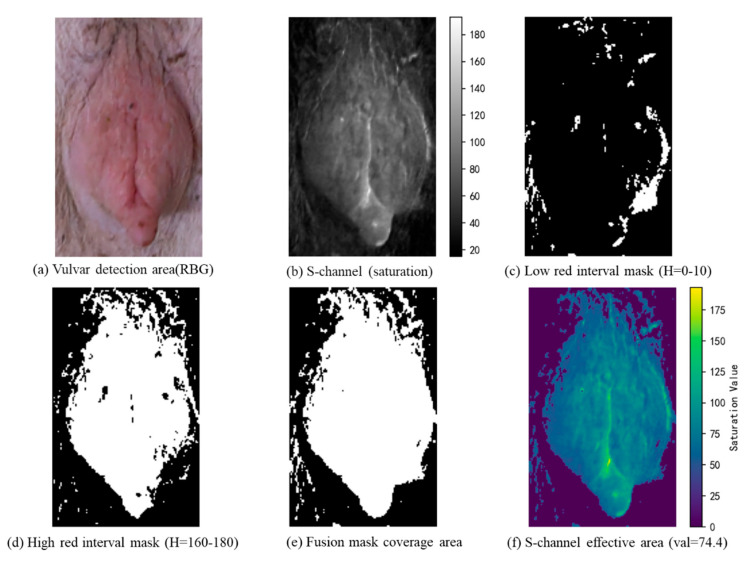
Extraction of vulvar congestion features based on a dual-threshold masking method.

The core advantage of this design lies in its multi-level optimization of feature robustness. By setting a saturation threshold (S ≥ 50), noise interference from shadows and other low-saturation areas is effectively filtered out, allowing the focus to remain on genuine congestion signals. Simultaneously, the use of two red intervals (H ∈ [0,10] and H ∈ [160,180]) fully covers the red spectrum—from deep red to magenta—thereby avoiding the color omission issues that may arise from a single threshold (see Figure 8). Furthermore, by calculating the mean saturation instead of relying on extreme values, the indicator reflects the overall congestion intensity of the vulva, reducing the impact of random fluctuations caused by localized blood stains or glare. This three-tiered strategy creates a progressive optimization approach that not only ensures the comprehensive extraction of physiological features, but also enhances the stability of the data representation.

In terms of aspect ratio feature extraction, the vulvar’s morphological features are directly calculated based on the detection box parameters:

(8) $$AspectRatio=\frac{h}{w}$$

The height (*h*) and width (*w*) are converted from the normalized coordinates output by the model into actual pixel values. Compared to directly measuring the absolute dimensions of the sow’s vulva [18,37], calculating the aspect ratio from the detection bounding box eliminates the influence of breed differences on the absolute size, using relative proportions to represent the degree of swelling while also mitigating the perspective distortion caused by variations in shooting angles or distances, thereby ensuring the objectivity of the morphological features.

This study constructs a bimodal indicator system by integrating both color and morphological features: red saturation quantifies the intensity of vascular dilation, and the aspect ratio characterizes the extent of tissue swelling. These two metrics reflect the estrus state from different physiological dimensions. Such a multi-dimensional feature extraction strategy not only enhances the biological interpretability of the detection indicators, but also provides discriminative criteria for distinguishing between congestion caused by inflammation and that induced by estrus.

#### 2.3.2. Lightweight MLP and Model Pruning

**Figure 9:**
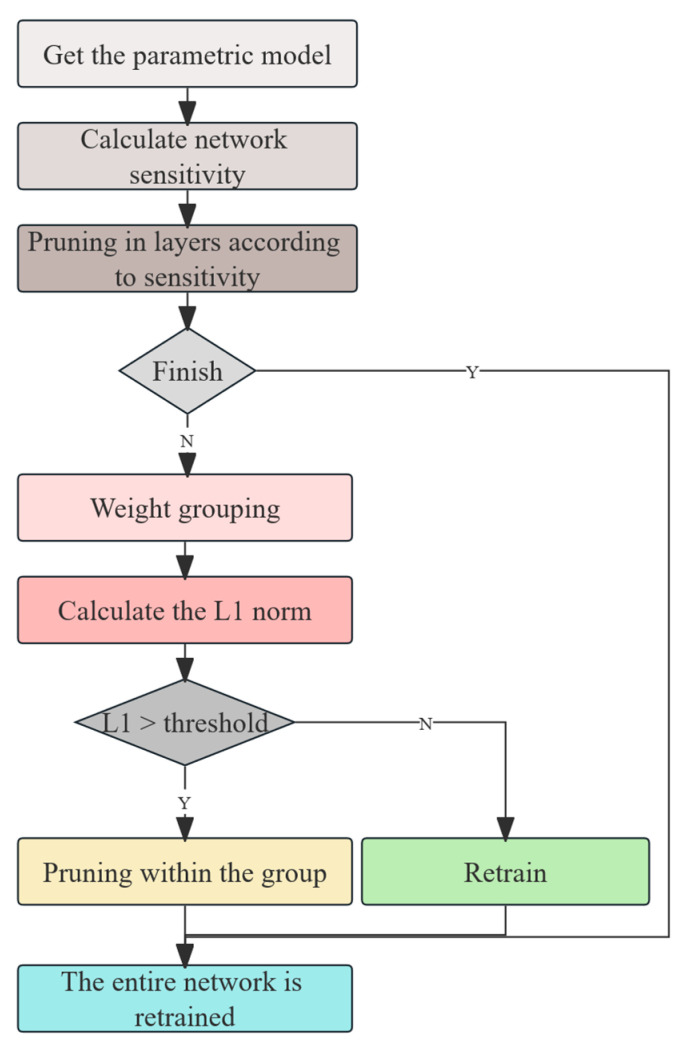
Model pruning process.

This study employs a lightweight MLP model to perform the binary classification task for determining the sow’s estrus state. An MLP is a specific type of feedforward neural network characterized by fully connected layers and the use of non-linear activation functions [38]. It consists of an input layer, one or more hidden layers, and an output layer, with each layer being fully connected to all neurons in the preceding layer. The input estrus feature data is subjected to weighted summation with an added bias, then transformed non-linearly through an activation function, and finally converted into a probability of estrus via a Sigmoid activation function [39] (see Figure 9). For the output $h_{l}$ layer, it can be expressed as follows:

(9) $$h_{l}=\sigma \left(w_{l}h_{l-1}+b_{l}\right)$$

where $h_{l-1}$ is the output from the previous layer; $w_{l}$ and $b_{l}$ are the weight matrix and bias vector of the l layer, respectively; and σ represents the ReLU activation function.

To further lighten the model, we applied L1 norm pruning to the MLP. L1 pruning is a method based on the absolute magnitude of the weights, evaluating the importance of each neuron or weight by calculating its L1 norm [40]. Parameters with relatively small absolute values are considered to contribute less to the model and can therefore be pruned, reducing both the computational load and storage requirements. The pruning process typically involves training the model, computing the L1 norms, pruning the weights based on a defined threshold, and then fine-tuning to recover any performance lost after pruning. L1 pruning is essentially based on the principle of the L1 norm to remove unimportant weight connections. The formula for L1 pruning is as follows:

(10) $$W^{pruned}=W\Pi \left(\left|W\right|>\lambda \right)$$

where *W* is the original weight matrix; Π is the indicator function that retains the weight only when its absolute value exceeds the threshold λ, and sets the weight to zero otherwise; and λ is the pruning threshold, typically determined based on the L1 norm ranking.

### 2.4. Deployment on Edge Devices and Applications

#### 2.4.1. System Hardware

**Figure 10:**
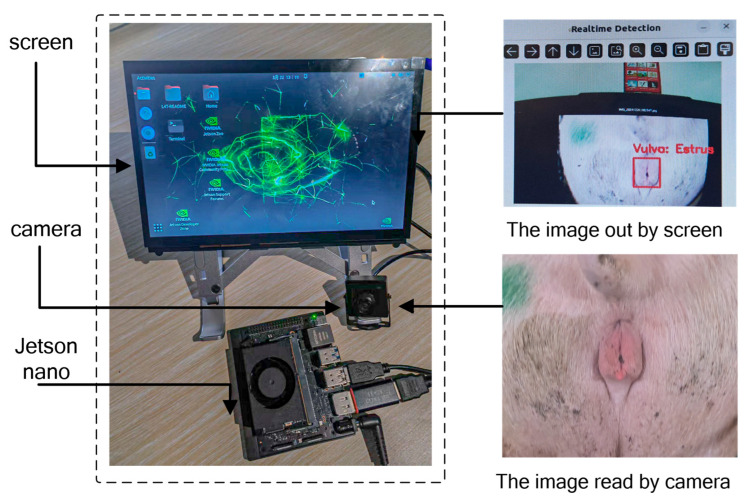
Estrus detection system equipment setup.

The system consists of a camera, a display, and a Jetson Nano edge computing device. The Jetson Nano is an NVIDIA embedded computing board powered by an NVIDIA Tegra processor, which integrates an ARM-based CPU and supports a wide range of AI frameworks. It is a low-power, high-performance system with power consumption as low as 5–10 W, and it delivers up to 472 GFLOPs of computing performance. With compact dimensions of 69.6 × 45 mm, the Jetson Nano offers excellent portability, making it ideally suited for the system designed in this study. Moreover, the camera and display are connected to the Jetson Nano via USB interfaces, facilitating easy device debugging and installation (see Figure 10).

#### 2.4.2. Overall System Design Workflow

First, the program initializes and loads the trained model. It then captures images of the vulvar region via the camera. The input video stream is decoded into individual frames, and these frames are preprocessed into a data format that can be directly accessed via the GPU. Next, the YOLOv11 detection module is executed to obtain the target recognition results for the sow’s vulva, including the bounding box coordinates. These coordinates are then provided to the estrus detection component, where the aspect ratio of the bounding box is used to determine the sow’s estrus state.

**Figure 11:**
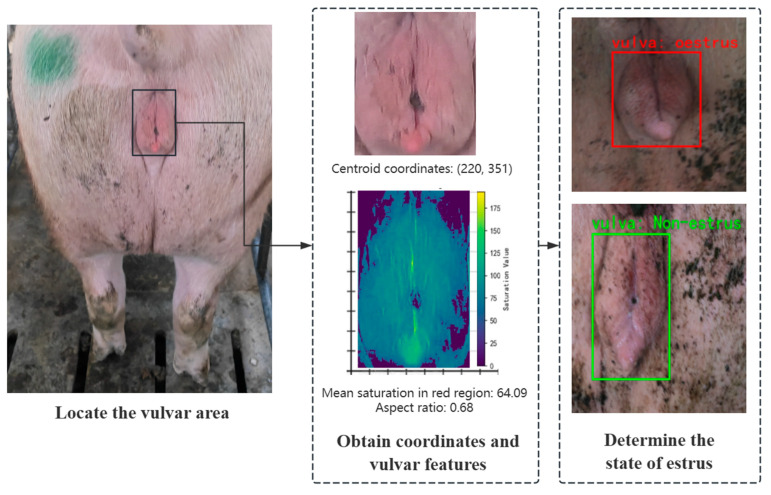
Estrus detection system workflow.

When the vulvar detection result indicates a non-estrus state, the bounding box and corresponding text are displayed in green with the label “Non-estrus.” Conversely, when estrus is detected, they are displayed in red with the label “estrus.” Additionally, to avoid misclassification due to a very small detected vulvar region, if the bounding box is too small, estrus detection is not performed, and a blue bounding box is used to prompt the operator to move closer (see Figure 11).

### 2.5. Evaluation Metrics

To comprehensively evaluate the performance of the proposed estrus detection system, we adopted metrics from both the object detection and classification domains. For vulva detection, we used Precision, Recall, and the mean Average Precision at an intersection-over-union threshold of 0.5 (mAP@0.5, or box mAP_50_). Precision reflects the proportion of correctly detected vulva regions among all detections and is defined as follows:

(11) $$Precision=\frac{TP}{TP+FP}$$

where TP denotes true positives and FP denotes false positives. Recall indicates the proportion of correctly detected vulva regions relative to all annotated ground-truth instances, given as follows:

(12) $$Recall=\frac{TP}{TP+FN}$$

where FN denotes false negatives. The mAP@0.5 was calculated as the mean area under the Precision–Recall curve across categories, where predictions are considered correct if the overlap between the predicted and ground-truth bounding boxes exceeds 0.5 IoU:

(13) $$IoU=\frac{\left|B_{p}\cap B_{gt}\right|}{\left|B_{p}\cup B_{gt}\right|}$$

These metrics together describe the localization accuracy of the detector.

For estrus classification, four indicators were applied: Accuracy, F1 score, AUC-ROC, and AUC-PR. Accuracy measures the overall proportion of correct predictions:

(14) $$Accuracy=\frac{TP+TN}{TP+TN+FP+FN}$$

but can be biased under class imbalance. To mitigate this, we used the F1 score, defined as the harmonic mean of Precision and Recall:

(15) $$F1=\frac{2\times Precision\times Recall}{Precision+Recall}$$

In addition, AUC-ROC evaluates the ability of the classifier to distinguish between estrus and non-estrus across varying thresholds, with values approaching 1.0 indicating strong discriminative power. AUC-PR, in contrast, focuses on the trade-off between Precision and Recall and is particularly informative when estrus cases are relatively scarce. By integrating detection-level and classification-level metrics, our evaluation framework ensures that both vulva localization and estrus status determination are rigorously assessed under realistic farm conditions.

## 3. Estrus Detection Results and Analysis

### 3.1. Training and Testing of the Target Detection Model

#### 3.1.1. Model Training

**Figure 12:**
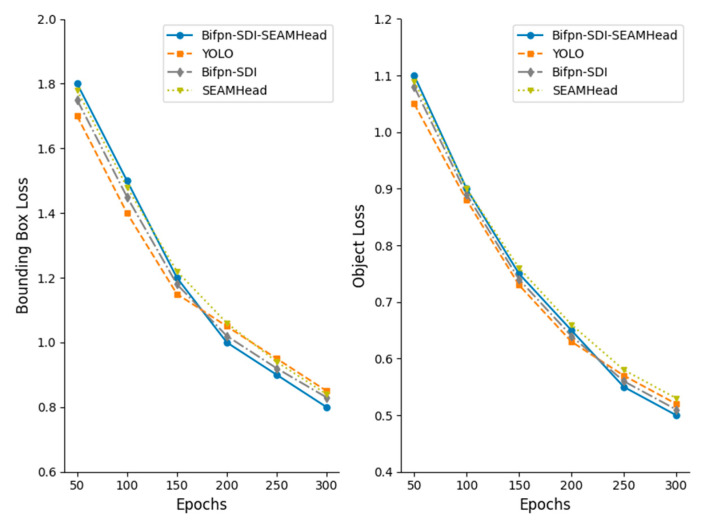
Bounding box loss and target loss curves.

After adjusting the network, the constructed dataset was fed into the optimized network for training, and batch image detection was employed to test the trained model. Prior to training, all network parameters were initialized. The training was conducted for 300 epochs, with a batch size of 16. The initial learning rate was set to 0.01, and a cyclic learning rate of 0.1 was used, with weights preloaded from YOLO11m.pt. Appendix A Table A1 shows the parameter configuration of the improved YOLO model during the runtime. The model saved the results at the end of each epoch and selected the best model for the vulva detection region. Figure 12 shows the trend of bounding box loss and target loss under different optimization strategies over 300 epochs. It can be observed that the model’s loss values gradually decreased and stabilized with increasing epochs. Notably, the model optimized with BiFPN-SDI-SEAMHead converged relatively faster, particularly during the final 100 epochs, where the loss decreased more rapidly compared to in other optimized models.

We adopted the Ultralytics default scheduler: a three-epoch linear warm-up from $1\times 10^{-4}$ to $1\times 10^{-3}$, followed by a cosine decay to $1\times 10^{-6}$ over the remaining 297 epochs. Where $\eta \left(t\right)$ is the learning rate of the t epoch, the closed-form expression is as follows:

(16) $$\eta \left(t\right)=\left\{\begin{array}{cc} 10^{-4}+9\times 10^{-4}\times \frac{t}{3}, & 0\leq t<3\\ 10^{-6}+\frac{1}{2}\left(10^{-3}-10^{-6}\right)\left[1+\cos\frac{\pi \left(t-3\right)}{297}\right], & 3\leq t<300 \end{array}\right.$$

As summarized in Table 2, by comparison, it can be observed that the model optimized with both SDI and SEAM achieved the highest precision, while the model optimized with SEAM alone outperformed the one optimized with SDI alone, due to SDI’s superior ability to extract vulvar features. The precision of the optimized models is significantly better than that of the original, unoptimized YOLOv11 model. Since subsequent measurements of the vulvar bounding box dimensions (length and width) will be performed, both the bounding box size (Box) and the detection accuracy (mAP_50_) are of critical importance.

As summarized in Table 3, the end-to-end latency on the Jetson Nano rig is 64 ms per frame (≈15.8 FPS), with 31 ms spent on pre-processing, 20 ms on inference, and 13 ms on post-processing. These measurements highlight that CPU-side resizing and color conversion dominate the overall runtime, suggesting that moving these steps to the GPU or using a pre-resized input stream would be the most effective ways to further increase the throughput.

#### 3.1.2. Comparison and Analysis of the Distilled Model

Table 4 compares the results of directly using the YOLOv11n model, the undistilled model, and the lightweight model after distillation. In terms of detection accuracy, the distilled lightweight YOLOv11m model achieved an mAP_50_ of 0.941, which is 1.1% lower than that of the undistilled model (0.952), but 11.8% higher than that of the YOLOv11n (0.823). This indicates that knowledge distillation effectively compensates for the performance loss associated with model lightweighting. Additionally, the distilled lightweight YOLOv11m model has a GFLOPs size of 6 G, representing a 91% reduction compared to the undistilled model, making it more suitable for deployment on embedded edge devices.

#### 3.1.3. Model Performance Testing

In this study, both video- and image-based methods were used to comprehensively evaluate the target detection performance. First, video data recorded on 19 December were selected to assess the model’s recognition capability. The model processes the video stream frame by frame using cv2, maintaining the original frame rate during detection. Each frame is resized according to a predefined scaling factor prior to being fed into the model to optimize the display effect. Although the per-frame inference in video may be influenced by the clarity of the video and the model’s inference time—potentially reducing the detection speed—it more closely reflects real-world application scenarios and better represents the operating conditions in pig farms.

The results in Table 5 show that, compared to the original YOLO model, the integration of BiFPN and SEAM-Head significantly improved the detection accuracy through multi-scale feature fusion and attention mechanisms. However, each one individually increases computational complexity: BiFPN extends the feature extraction time due to the generation of multi-level feature maps, while SEAM-Head’s attention computations add to the per-frame processing time. When used separately, both modules can optimize the feature storage to reduce the memory usage; however, when combined, the surge in the number of feature maps leads to an increased computational load from SEAM-Head, causing the overall memory consumption and computational demand to rise concurrently. By applying knowledge distillation, the model size is compressed while retaining accuracy—the student network significantly reduces the number of parameters, feature maps, and computational branches, as well as the storage of activation values and the requirements for gradient computation. Ultimately, the distilled model is optimized in terms of storage space, runtime memory, and per-frame detection time, striking a balance between accuracy and efficiency and making it better suited for real-time detection on edge devices.

**Figure 13:**
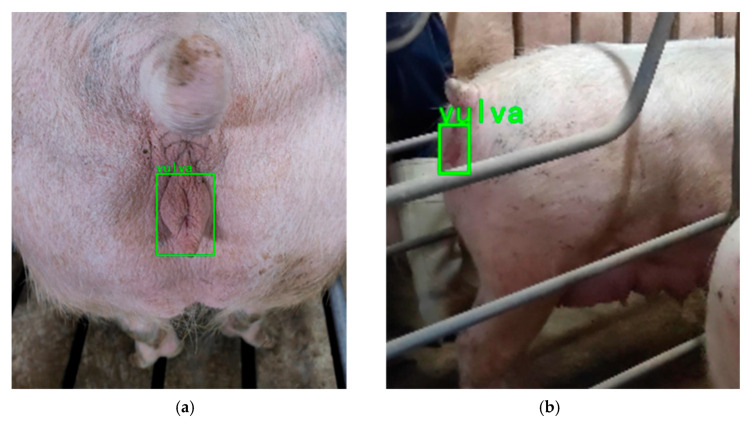
Detection results of sow vulvas from different angles: (**a**) sow vulva frontal detection results; (**b**) sow vulva lateral detection results.

To further evaluate the model’s annotation capability under different environmental conditions, this study tested the model using 60 untrained images. At the same time, in order to test the model’s ability to detect small and fuzzy objects, this includes unclear vulvar images from the front and sides. As shown in Figure 13, the method presented in this study not only accurately identifies the sow’s vulvar region against complex backgrounds, but also precisely recognizes vulvar regions of various sizes and from various angles. It can be observed that the proposed method is effective in marking the sow’s vulva across different shooting conditions.

### 3.2. Analysis of the Relationship Between Vulvar Features and Estrus State

#### 3.2.1. Analysis of Vulvar Features in Estrus Sows

This study statistically analyzed the vulvar features of sows during estrus and non-estrus periods, as shown in Table 6, focusing on the aspect ratio (AR) and color saturation. A stratified random subset of 498 images (148 estrus; 350 non-estrus) was used for statistical comparison, in order to avoid an excessive leverage of repeated images from the same animal.

Both metrics violated normality (SW *p* < 0.05); the aspect ratio also violated homoscedasticity (Levene *p* < 0.05). Therefore, two-sided Mann–Whitney U tests were applied (n_1_ = 148, n_2_ = 350), yielding U = 826 (r = 0.97) for the aspect ratio and U = 49,905 (r = 0.92) for saturation. For completeness, we also report the parametric effect sizes and confidence intervals: the 95% CI of the mean difference in the aspect ratio was [−0.357, −0.288], with Cohen’s d = 1.23; for saturation, the 95% CI was [18.56, 22.26], with Cohen’s d = 2.23.

The aspect ratio was markedly lower in estrus sows than in non-estrus sows (U = 826, *p* < 0.001), indicating that the vulvar outline becomes more circular during estrus. The color saturation was conversely much higher in estrus animals (U = 49,905, *p* < 0.001), reflecting pronounced vascular congestion and a deeper red hue. The rank-biserial effect sizes were r = 0.97 for the AR and r = 0.92 for saturation, both considered “very large”, confirming that the two features are highly discriminative for estrus detection.

#### 3.2.2. Evaluation of Classifier Effectiveness for Estrus Detection

As shown in Table 7, directly applying YOLO for estrus classification yielded poor results, with an Accuracy of only 0.58 and a Precision of 0.56. This demonstrates that YOLO alone is inadequate for reliable estrus recognition, as it often misclassified illumination changes or vulva occlusions as estrus signals. Even after optimization, the YOLO-based direct classification remained suboptimal, suggesting that improvements in detection modules alone cannot address the limitations of end-to-end classification.

In light of this limitation, we further investigated several commonly used classifiers—Support Vector Machine (SVM), Random Forest (RF), and Multi-Layer Perceptron (MLP)—by using extracted vulvar features as inputs. Comparative experiments demonstrated that MLP achieved the best performance (Accuracy = 0.91, Precision = 0.90), surpassing both SVM and RF. This performance is attributed to MLP’s ability to capture complex non-linear feature relationships and generate stable predictions in binary classification tasks.

Taken together, these findings confirm that estrus detection requires a two-stage pipeline in which YOLOv11 is used for vulva region localization, while a dedicated classifier is employed for estrus state recognition. Among the tested classifiers, MLP not only delivered the most robust and accurate results, but also integrated effectively with YOLOv11, enabling a practical and interpretable solution for deployment on edge devices.

#### 3.2.3. Construction of the Estrus Detection Model Based on a Lightweight MLP

**Figure 14:**
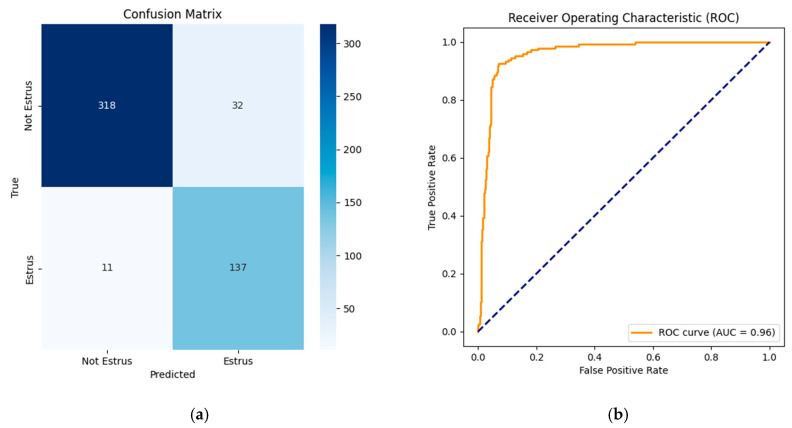
Lightweight MLP prediction results: (**a**) confusion matrix; (**b**) AUC-ROC curve; and AUC-ROC: Area Under the ROC/Precision–Recall Curve. The diagonal dashed line represents the performance of a random classifier (AUC = 0.5) as a reference baseline.

**Figure A1:**
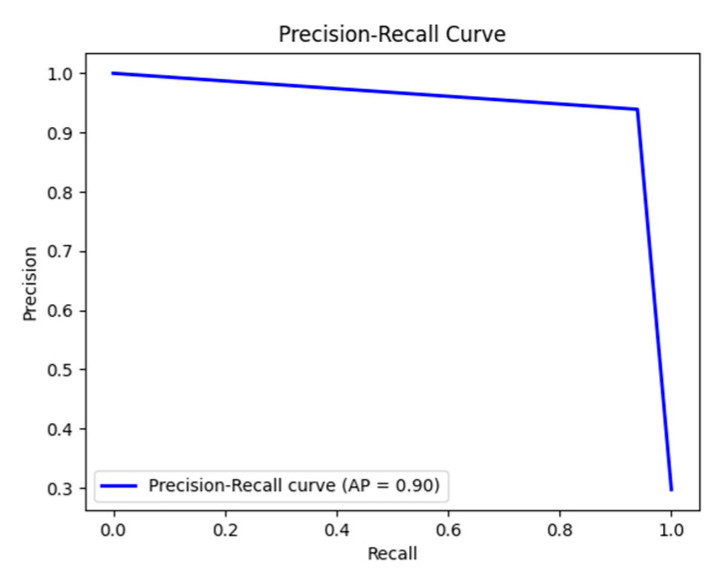
PR curve.

The extracted data on the sow’s vulvar aspect ratio and the proportion of red region saturation were normalized, and a lightweight MLP network was then trained to establish an estrus prediction model based on vulvar features. After multiple training iterations, the model achieved optimal accuracy at 300 epochs. Finally, the model’s predictions were compared with the actual estrus conditions. The proposed model achieved an AUC-ROC of 0.96, demonstrating an excellent classification capability. It can be seen from Figure 14, Table 8 and Appendix A Figure A1 that the model obtained favorable results, with an F1 score of 0.86, an Accuracy of 0.91, and an AUC-PR of 0.86, while the area under the Precision–Recall curve (AUC-PR) reached 0.90, indicating that the changes in the aspect ratio and color features during estrus exhibit consistent patterns.

### 3.3. Estrus Detection Model System Testing

To further evaluate the effectiveness of the optimizations and the practical application capabilities on edge devices, video data were imported into the Jetson device for field experiments. The system was tested for identifying non-estrus and estrus, with the test results illustrated through images. The results demonstrate that the model is capable of accurately marking the sow’s vulvar region and reliably indicating the estrus state.

**Figure 15:**
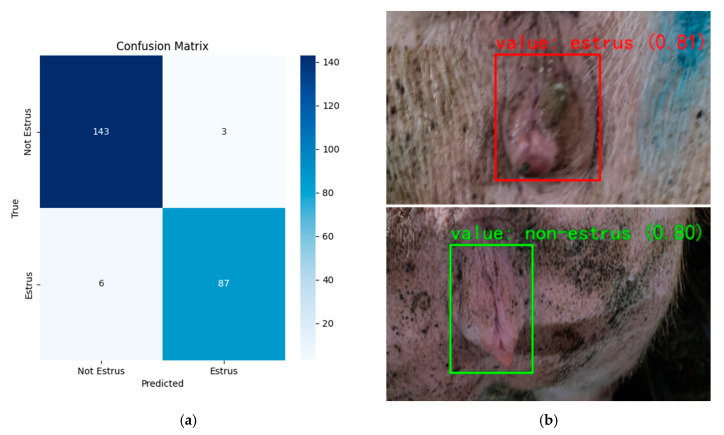
Pig farm test results: (**a**) confusion matrix; (**b**) vulva test results (estrus/no estrus).

This study collected sow estrus detection results from the farm and compared them with the assessments carried out by researchers on the same day. In this actual scenario, a total of 250 sows were tested. As shown in the confusion matrix of Figure 15a, the overall Accuracy of the model is 96.2%, the Precision is 96.7%, and the Recall is 93.5%, which is highly accurate. At the same time, Figure 15b shows that the detection effect of the model is clear and the markings are clear. It demonstrates its strong ability to identify estrus cases in the actual pig farm environment, proving that it is capable of performing pig farm detection tasks.

By analyzing the false negative estrus cases, we found that occlusion, lighting, and blur are the main factors causing this situation. Possible solutions include adding an automatic white balance module, deploying a side auxiliary camera to reduce occlusion, and applying temporal smoothing to consecutive frames. These improvements will be implemented in our subsequent study.

## 4. Discussion

The present study demonstrates that our improved YOLOv11-BiFPN-SDI-SEAMHead detector, combined with a lightweight MLP classifier, enables accurate and interpretable estrus prediction based on vulva localization and feature fusion. Through model enhancements—including BiFPN-SDI and SEAM-Head modules—and mask-guided knowledge distillation, we achieved ~91% model compression (from 67 GFLOPs to 6 GFLOPs, ~3.96 MB) while maintaining high detection accuracy (mAP_50_ ≈ 0.94, AUC ≈ 0.96) and real-time performance (~16 fps) on a Jetson Nano.

### 4.1. Evaluation of the Estrous Detection Models

As summarized in Table 9, previous studies have explored different sensing and inference strategies. Texture-based methods using GLCM and neural networks [18] offered limited accuracy (~70%) and lacked real-time capability. YOLOv4-based size estimation [19] achieved high object detection performance (>97%), but required geometric calibration and did not include any estrus classification mechanisms, limiting its practical utility. Robotic imaging approaches with 3D vulva volume reconstruction and 1D-CNNs [20] demonstrated strong test accuracy (~98%) by integrating behavior and structural features, but their reliance on LiDAR sensors and overhead robotic platforms substantially increases system complexity and deployment cost. Infrared-based systems such as Xue et al.’s [21] combined YOLOv5s-based segmentation with ensemble regression, to predict the rectal temperature from vulva thermal images, achieved high precision (MSE = 0.114 °C and IoU = 91.5%). However, their study did not address estrus detection or classification; instead, it focused solely on non-contact physiological monitoring. The segmentation and regression components were functionally separate, and no model was trained to infer the estrus status.

In contrast, our approach directly extracts biologically grounded morphological and color features (aspect ratio and red saturation) from RGB images, enabling efficient estrus classification using low-cost, scalable hardware. The system runs in real time on edge devices without the need for sensor calibration, 3D reconstruction, or behavior fusion, offering a practical balance of accuracy, interpretability, and deployment feasibility for commercial sow estrus monitoring.

This study also found that YOLO alone is insufficient for reliable estrus classification. While the modified YOLO module enhanced the localization of the vulva region, it failed to capture subtle physiological signals. In contrast, combining biologically meaningful features with a lightweight MLP classifier significantly improved accuracy and interpretability, confirming the necessity of a two-stage design.

### 4.2. Current Deficiencies and Future Studies

**Figure 16:**
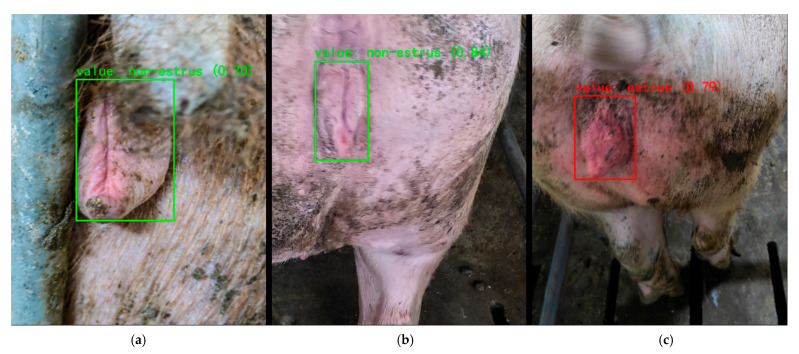
Representative failure cases of estrus detection: (**a**) occlusion; (**b**) blur; and (**c**) inflammation.

Figure 16 shows the test results. Despite the overall strong performance, the system still encounters some failure cases under challenging conditions. The partial occlusion of the sow’s vulva or motion blur in the images can cause the detector to miss or mis-localize the target, thereby reducing the recognition accuracy. In addition, if the vulva’s red coloration is not pronounced due to suboptimal lighting or limited camera sensitivity, the color cue that the classifier relies on is weakened, which may lead to missed estrus detections. Certain non-estrus physiological conditions such as inflammation or postpartum changes can also cause vulvar swelling or redness that the model might mistakenly interpret as estrus.

While the optimized model performed well in our tests, it still faces certain limitations and challenges in more complex, real-world farm environments. First, because the training data were collected under relatively uniform conditions, the model’s generalizability to different breeds, group housing systems, and variable lighting conditions remains to be verified. Second, vulvar changes caused by factors unrelated to estrus—for example, infections or injuries—could confuse the feature-based diagnosis, indicating the need for the more robust discrimination of estrus-specific cues. To further enhance the system’s robustness, future work will expand the diversity of the dataset by incorporating sows of various breeds, ages, and housing conditions, and will explore multi-modal data fusion to mitigate the effects of lighting variations and occlusion. We also plan to incorporate temporal analysis by tracking the same sow’s vulvar changes across multiple estrus cycles, which could help to distinguish true estrus signals from anomalous variations over time. Addressing these issues through cross-modal integration and longitudinal monitoring will improve the model’s versatility and reliability, paving the way for deployment in more complex farm settings.

## 5. Conclusions

In this study, we proposed a lightweight and deployable sow estrus detection system based on an improved YOLOv11 detector and biologically meaningful vulvar features. By introducing the BiFPN-SDI module and SEAM-Head attention mechanism, our model significantly enhanced vulva localization accuracy while maintaining real-time performance on resource-constrained edge devices through knowledge distillation. Furthermore, the system uses the aspect ratio of the detected vulva and the red saturation in the HSV color space as key features and applies a lightweight MLP classifier to determine the estrus status. This biologically grounded feature combination ensures both accuracy and interpretability in classification, with an AUC-ROC of 0.96 and Accuracy exceeding 91%. Field validation demonstrated that the system achieved 82% Recall in practical farm conditions, with a low-cost hardware setup, highlighting its economic viability compared to thermal or LiDAR-based alternatives. These findings provide a feasible and scalable solution for intelligent sow estrus monitoring and lay the foundation for future integration with autonomous farm inspection platforms.

## 6. Patents

The work presented in this manuscript is related to the patent of “Sow Estrus Detection Method, Device, Equipment, Medium, and Computer Program Product (Application No. CN120071388A)”, which is currently under substantive examination. No licensing fees, royalties, or equity interests have been generated to date.

## Figures and Tables

**Table 1:** Model complexity calculation.

Model	Stages	Sampling	Head	Params (M)	FLOPs (M)	Latency (ms)
BiFPN-SDI	5 × SDI	3 + 2	4	0.12	3.1	0.40
Detect_SEAM	3 × 1 × 1 Conv	1	—	0.066	1.4	0.28
**Total**	—	—	—	**0.19**	**4.5**	**0.68**

**Table 2:** Ablation study of different optimization algorithms.

No.	BiFPN-SDI	SEAMHead	Box	R	mAP_50_
1			0.838	0.789	0.893
2	✓		0.894	0.787	0.903
3		✓	0.911	0.826	0.943
4	✓	✓	0.933	0.846	0.952

mAP_50_: mean Average Precision at IoU 0.5; Box: bounding box size metric; and R: Recall.

**Table 3:** Runtime breakdown of the sow estrus detection pipeline on the Jetson Nano.

Metric	Value
Processed frames	324
Avg pre-processing latency (ms/frame)	31
Avg inference latency (ms/frame)	20
Avg post-processing latency (ms/frame)	13
Total pipeline latency (ms/frame)	64
End-to-end FPS (frames/s)	15.8

**Table 4:** Comparison of distilled model accuracy.

Model	Box	R	mAP_50_	GFLOPs
YOLOv11n	0.824	0.768	0.823	6.1 G
YOLOv11m	0.933	0.846	0.952	67.0 G
YOLOv11m(Lightweight)	0.923	0.837	0.941	6.0 G

GFLOPs: Giga floating-point operations per second.

**Table 5:** Comparison of models’ computational performance under different optimization effects.

No.	BiFPN-SDI	SEAMHead	Lightweight	Storage Size (MB)	Detection Time (ms)	Running Memory (MB)
1				5.07	16.12	1239.84
2	✓			32.48	27.58	1238.55
3		✓		38.54	25.82	1234.11
4	✓	✓		32.38	24.29	1245.11
5	✓	✓	✓	3.96	18.87	1238.09

**Table 6:** Statistical analysis of sow vulvar features.

	Group Mean ± SD					
Metric	Non-Estrus	Estrus	Shapiro–Wilk *p*	Levene *p*	Test Used (Stat)	*p*
AR	0.973 ± 0.314	0.682 ± 0.272	1.6 × 10^−6^/2.2 × 10^−26^	4.8 × 10^−7^	U = 826	<0.001
Saturation	73.621 ± 11.661	87.010 ± 9.777	4.0 × 10^−8^/4.8 × 10^−15^	0.239	U = 49,905	<0.001

AR is a shorthand for aspect ratio; SW = Shapiro–Wilk test; and *p* values shown as estrus/non-estrus.

**Table 7:** Comparative evaluation of classifier and detection strategies for sow estrus detection.

Detection Method	Accuracy	Precision
YOLO (Direct classification)	0.58	0.56
Improve YOLO (Direct classification)	0.72	0.60
Improve YOLO + SVM	0.89	0.86
Improve YOLO + RF	0.87	0.89
**Improve YOLO + MLP**	**0.91**	**0.90**

**Table 8:** Results of various statistical indicators.

Statistical Indicators	Numeric Value
Accuracy	0.9137
F1 Score	0.8644
AUC-ROC	0.9600
AUC-PR	0.8618

**Table 9:** Comparison of different solutions.

Feature Type	Analysis Method	Performance	Hardware	Inference Speed	Deployment Feasibility	Interpretability	Reference
Texture	BPNN	Accuracy ≈ 70%	RGB camera	\	No	Texture features	[18]
Vulva size	YOLOv4	Accuracy > 97%	RGB camera	≈2.8 fps	No	Size directly meaningful	[19]
3D volume	LiDAR + 1D-CNN	Accuracy = 92.3% ± 10.1%	LiDAR + RGB + Robot	\	No	Biologically grounded	[20]
Vulva temperature	YOLOv5s	Segmentation IoU = 91.5%	RGB camera	≈49 fps	No	Temperature cues	[21]
Aspect ratio and red saturation	Optimizing YOLOv11 + MLP	AUC = 0.96, Accuracy ≈ 91.4%	RGB camera + Jetson Nano	≈16 fps	Yes	Physiologically interpretable	This study

Reference [19] is the vulva target and size recognition rate and ref. [21] is the vulva target recognition rate.

## Data Availability

The data are available upon request due to restrictions (e.g., privacy or legal or ethical reasons). Since the information of the pig farm has a certain commercial nature, it cannot be disclosed without the permission of the pig farm.

## Abbreviations

The following abbreviations are used in this manuscript:

AR	Aspect ratio
MLP	Multilayer Perceptron
SVM	Support Vector Machine
RF	Random Forest
MGD	Masked Generative Distillation
BiFPN	Bi-directional Feature Pyramid Network
SEAM-Head	Semantic Enhancement Attention Module
mAP	mean Average Precision
IoU	Intersection over Union

## Appendix A

**Table A1:** Key hyperparameters of the YOLOv11-BiFPN-SDI–SEAM process.

Category	Hyperparameter	Value
YOLOv11 backbone	Depth–width scale	0.50 depth, 1.00 width
	Input resolution	640 × 640 px
	Max channels	512
BiFPN-SDI neck	Multi-scale levels	P3–P7 (5 levels)
	SDI attention heads	4
SEAM-Head	Channel reduction ratio *r*	16
MLP estrus classifier	Hidden layer sizes	[32, 16]
Knowledge distillation (MGD)	Loss weight λ	0.5
	Mask ratio	0.25
L1 pruning	Threshold λ	0.0005
	Pruned percentage	30%
Training settings	Learning rate schedule	0.0001 → 0.001
